# Violence, misconduct and schizophrenia: Outcome after four years of optimal treatment

**DOI:** 10.1186/1745-0179-1-3

**Published:** 2005-04-28

**Authors:** Marina Economou, Alexandra Palli, Ian RH Falloon

**Affiliations:** 1 Department of Psychiatry, University of Athens, Greece; 2 Department of Psychiatry, University of Auckland, New Zealand

**Keywords:** aggression, sexual misconduct, schizophrenia, evidence-based treatment, prospective evaluation

## Abstract

**Background:**

Aggressive behaviour in patients with schizophrenic disorders is an ongoing source of concern to community-based services. It has been suggested that optimal treatment may reduce the risk of serious misconduct.

**Objective:**

To assess prospectively aggressive and sexual misconduct in a cohort of patients receiving continued evidence-based community treatment.

**Method:**

Fifty patients with a DSM-IV diagnosis of a schizophrenic disorder were treated for 4 years with integrated biomedical and psychosocial strategies. The frequency and context of all aggressive and sexually inappropriate behaviour were assessed throughout. Correlations between an index of misconduct and demographic and clinical variables were examined.

**Results:**

Levels of serious misconduct were low at the start of the project and declined as treatment progressed. Close examination of predictors of misconduct supported larger epidemiological studies imputing persistent psychotic symptoms, personality disorders and substance use.

**Conclusion:**

The study supports the hypothesis that effective treatment reduces aggressive and sexual misconduct in schizophrenic disorders.

## Introduction

Current concerns about community care of the mentally disordered focus on the level of risk of unacceptable behaviour, particularly inappropriate aggressive or sexual acts. It is clear that such behaviour occurs in patients suffering from mental disorders, and might be expected to be more common in psychotic states, when provocation from delusional and hallucinatory perceptions is present [1-7]. The presence of concurrent substance abuse, personality disorder or history of previous violent or antisocial behaviour increases the risk of people with psychotic disorders committing violent offences [1,2,8-10]. The methodology of most of theses studies on which these conclusions are based has been flawed, with selection bias, lack of diagnostic precision and results often based upon criminal records rather than clearly defined ratings of behaviours [3,11-14]. The issue of unreported and non-criminal aggressive behaviour, including inappropriate sexual behaviour, particularly in the context of family or residential care has been studied less extensively [15,16]. Furthermore the course of this misconduct and the effects of continued community treatment have been seldom examined. One excellent study compared the course of aggressive behaviour in a cohort of patients after discharge from acute psychiatric hospital care [17]. In one center a comparison group of people living in the same neighbourhoods indicated that after the initial 10 weeks of follow-up there was no difference in the prevalence of violence by patients and that of their neighbours, when substance abuse was absent in both groups. Violence in both groups was usually committed at home and targeted at family and friends. The context of this violence was not explored in detail, although it is evident that severely mentally disordered patients are often themselves the victims of violent behaviour [18,19]. It is probable that a substantial proportion of aggressive acts may be provoked by aggression and coercion towards the patients themselves. Finally, although it is clear that psychoactive medication can induce a substantial reduction in aggressive behaviour [20,21], and that non-adherence to community-based treatment is a predictor of severe violence in schizophrenia [10,22] the effects of long-term optimal treatment programmes on the level of aggressive behaviour have seldom been reported.

It may be concluded that the hypothesis that aggressive and sexually inappropriate behaviour is more common in people suffering severe schizophrenic disorders, particularly those characterised by persisting psychotic symptoms, with impaired decision-making abilities, including difficulties with adherence to community-based treatment programmes, has not been proven conclusively. There appears to be an increased risk of misconduct in cases when active psychotic experiences provoke anger and frustration, particularly when judgement is further clouded by drugs or alcohol [11]. There is an urgent need to provide reassurance to the community that efforts to prevent offensive behaviour by people with schizophrenia are realistic and that offending is managed according to the same standards of justice expected of all citizens.

The present report describes the changes in a broad range of aggressive behaviour and sexual misconduct in a cohort of chronic patients who were receiving continued evidence-based biomedical and psychosocial treatment throughout a four-year period. It was hypothesized that continued treatment would contribute to a lower rate of these behaviours over time.

## Methods

### Selection of cases

51 consecutive cases of schizophrenia with a DSMIV diagnosis of schizophrenic disorder based on a SCID-IV interview who were resident in central Athens and receiving continued treatment at the Kessariani Community Mental Health Service. These cases were a cohort who consented to participate in a 5-year study of the clinical and social outcome of continued evidence-based treatment pharmacological and psychosocial strategies [23].

### Assessments

#### OTP Misconduct Checklist

All aggressive behaviour and sexual misconduct was noted on a continual basis over a four-year period. Each year a standardised rating was made by independent assessors, who interviewed staff, patients and key caregivers to ascertain the frequency of nine varieties of misconduct: shouting, verbal and non-verbal threats, pushing, slapping, punching, use of a weapon, inappropriate sexual behaviour, sexual assault, and aggression towards property or animals. The frequency ratings varied from 0 = not at all; 1 = 1–10 times a year; 2 = more than 10 times a year; 3 = at least once a week. Where misconduct was reported the context of the behaviour was reviewed and a rating of provoking circumstances was made: 1 = when clearly provoked; 2 = when mildly provoked; 3 = unprovoked. Inter-rater reliability on 50 co-rated assessments ranged from ICC = 0.78 to 0.95 on the frequency items and 0.71 to 0.89 on the contextual ratings. Two items were not scored on any of the cases. These were use of a weapon and sexual assault.

### Background, social and clinical variables

A standardized structured assessment of all socio-demographic and clinical variables was made at 0, 12, 24, 36 and 48 months. This included age, gender, ethnicity, marital status, living situation, education, employment, duration of disorder, course of disorder, multi-axial diagnosis, clinical impairment, social disability, caregiver stress, social support, cooperation with treatment, justice system involvement, days in gaol, treatment received, and side effects of medication.

### Data analysis

Frequency counts and percentages were used to present descriptive data. Non-parametric correlations were conducted with Spearman's Rho to examine associations between clinical and social measures and levels of misconduct at each assessment point.

The **Index of Misconduct **was a weighted score that accounted for the nature of the misconduct, the degree of provocation as well as its frequency. Standardised scores ranged from 0–3 (**0 = no misconduct reported; **1 = **mild**, infrequent or clearly provoked verbal aggression; **2 = moderate**; frequent or unprovoked verbal aggression or any act of physical or sexual aggression; **3 = severe: **repeated physical or sexual aggression, often accompanied by verbal aggression).

## Results

The cohort is described in Table1. There were similar proportions of men and women aged between 20 and 50 years, almost all of whom were unmarried. Most (87%) were living with their parents of other family members. Thirty-one percent had not completed high school education, and two cases had mild mental retardation. The average duration of mental disorders was 14 years, with 2 cases experiencing their first episodes of schizophrenia. On average they had been admitted to hospital 3.3 times. More than half had persistent positive (55%) and negative (58%) symptoms at the beginning of the study. Nearly a third were considered to have a personality disorder, but very few used alcohol or drugs excessively. One fifth experienced moderate or severe side effects of their medication. Almost a quarter (23.5%) had some problems cooperating with their treatment programs.

**Table 1:** Description of the cohort of 51 cases of schizophrenic disorders

Age: mean years (s.d.)	35.4 (6.9)
Gender: male (%)	25 (49)
female (%)	26 (51)

Marital status	
single (%)	46 (90.2)
married (%)	1 (2.0)
separated/divorced (%)	4 (8.8)

Diagnosis:axis 1 DSM-IV	
paranoid (%)	21 (41.2)
disorganized (%)	5 (9.8)
catatonic (%)	1 (2.0)
residual (%)	8 (15.7)
undifferentiated (%)	5 (9.8)
schizoaffective (%)	11 (21.6)

Axis 2:	
personality disorder	16 (31.5)
mild mental retardation	2 (3.9)

Substance use	
none	36 (70.6)
within cultural norms	5 (9.8)
likely to cause health or social problems	4 (7.8)
likely to cause mental health problems	6 (11.8)

Side effects of medication	
none	25 (49.0)
mild	13 (25.5)
moderate	7 (13.7)
severe	3 (5.9)

The proportions of subjects who behaved frequently (10 or more times a year) in an aggressive or sexually inappropriate manner are summarized in Table 2. Shouting was the most common aggressive behaviour. At the beginning of the study this was reported in one-fifth of cases, followed by verbal threats in one-sixth. Physical and sexual aggression was displayed by less than 10% of cases and in no instance was a weapon of any sort used. Two thirds of cases showed no misconduct of any sort in the year preceding the initial assessment.

**Table 2:** Frequent or Very Frequent verbal, physical and sexual aggressive behaviour in 51 outpatients with schizophrenic disorders in the year before and throughout 4 years of evidence-based treatment

REPORTED BEHAVIOUR	Year 00 (%)	Year 01 (%)	Year 02 (%)	Year 03 (%)	Year 04 (%)
Shouting	11 (21.5)	8 (15.7)	7 (13.7)	5 (9.8)	5 (9.8)
Verbal threats	9 (17.6)	7 (13.7)	4 (7.9)	4 (7.8)	4 (7.8)
Pushing	4 (7.8)	2 (3.9)	0 (0.0)	1 (2.0)	1 (2.0)
Slapping	2 (3.9)	2 (3.9)	0 (0.0)	0 (0.0)	0 (0.0)
Punching	1 (2.0)	2 (3.9)	0 (0.0)	0 (0.0)	0 (0.0)
Use of weapons	0 (0.0)	0 (0.0)	0 (0.0)	0 (0.0)	0 (0.0)
Inappropriate sexual behaviour	2 (4.0)	1 (2.0)	1 (2.0)	0 (0.0)	0 (0.0)
Aggressive sexual behaviour	0 (0.0)	0 (0.0)	0 (0.0)	0 (0.0)	0 (0.0)
Other antisocial acts	4 (7.9)	3 (5.9)	1 (2.0)	0 (0.0)	0 (0.0)
Days in gaol	0	0	0	0	0
At least one aggressive behaviour	18 (35.3)	14 (27.5)	13 (25.5)	13 (25.5)	12 (23.5)
Moderate to high index of misconduct	11 (21.6)	6 (11.8)	3 (5.9)	3 (5.9)	3 (5.9)

The Index of Misconduct that weighted the severity of aggressive behaviour, both the nature of the acts as well as the presence or absence of provoking circumstances, was at least moderate at the baseline assessment in one-fifth of cases.

It is evident that the number of cases showing aggressive and sexual misconduct declined throughout the course of the 4 years of optimal treatment. At the end of this period the number of cases showing frequent aggressive behaviours had halved. More than three-quarters showed no aggressive or sexual misconduct at all, and the index of misconduct had declined from a mean of 0.67 (± 1.03) to a mean of 0.33 (± 0.71), with only 3 cases showing persistent pattern of aggressive behaviour after the first year of the program.

Involvement with the criminal justice system was negligible.

### Predictors of Misconduct

The social, demographic, symptom pattern, impairment, disability, caregiver stress, cooperation with the treatment program, medication side effects did not correlate with the index of misconduct at baseline or any other assessment points. The severity of disability (Spearman's Rho = .285, p = .042), and use of non-prescription substances (Rho = .280, p = .047) and cooperation with treatment (Rho = .313, p = .025) were associated with the misconduct index at baseline. The GAF score (Rho = -.295, p = .036) was the only variable that showed a significant correlation with misconduct in the first year of treatment. Caregiver stress was consistently, although not significantly, associated with the severity of misconduct over the 4 years of treatment with coefficients ranging from .256 (p = .076) in year one, .352 (P = .015) in year two, .273 (p = .064) in year 3 and Rho = .281 (p = .056) in the fourth year. In the fourth year GAF (Rho = -.315, p = .024) and the impairment rating (Rho = .283, p = .044) were significantly correlated with misconduct.

Given the low number of subjects and the modest associations between variables no attempts were made to use multivariate analyses. However, there were no trends towards association between misconduct and gender, persisting positive or negative symptoms, or medication side effects.

Close examination of the 21 cases with a paranoid syndrome diagnosis suggested that this symptom profile might predict moderate or severe misconduct. In the year before the study 33% (7/21) of cases with a paranoid syndrome fell into this category, compared to 13% (4/30) with non-paranoid syndromes. In subsequent years the number of cases with moderate/severe misconduct fell to minimal levels, but two of the three persistent cases had a paranoid symptom pattern.

Personality disorders that have been associated with misconduct include antisocial, borderline, paranoid, narcissistic and histrionic. We compared this group with those with other personality disorders, or those who did not have a DSM-IV axis two syndrome. In the year before the study 67% (6/9) of cases with a diagnosis of these personality disorders fell into the moderate or severe misconduct category in contrast to 12% (5/42) of those who did not have these vulnerability features. Again, two of the three persistent cases were in this personality disorder subgroup, and the third had a very early onset schizo-affective disorder with a schizoid personality disorder and persisting anxiety.

## Discussion

The present study is the first that we are aware of that examined the pattern of aggressive and sexually misconduct of patients with a diagnosis of schizophrenia over a period of several years during which time all subjects were receiving evidence-based treatment, with high levels of adherence. Although the sample was small, complete data was obtained on all cases.

The most remarkable finding was a low rate of physically violent behaviour. Only 7.9 per cent of the patients were reported as having slapped or punched another person within the year before the study, and after four years this had reduced to 3.9 per cent. None of these acts had been reported to the justice system. This rate of violent behaviour is contrasted with substantially higher rates of similar misconduct in the US, UK and Scandinavia [3,13,15] However in the absence of a matched comparison group of people with similar educational and social background suffering similar levels of disability and discrimination, but without schizophrenic disorders, the low rates of misconduct in this sample cannot be used to draw any specific conclusions. Similarly the lack of a matched treatment control group prevents us drawing definitive conclusions that may link the progressive reduction of misconduct to effective treatment.

Despite these limitations, several issues are worthy of further discussion. First, it should be noted that serious violence or offensive behaviour among psychiatric patients seems rare in Greece. There is a complete absence of secure units or units specializing in criminal violence anywhere in Greece. There is also a widespread belief amongst psychiatrists that although some patients are violent this is seldom severe. Secondly, it may be noted that only 12% of the sample used any substances that might have exacerbated their mental symptoms and contributed to behavioural problems. This relatively small group included those drinking excessive coffee or other caffeinated drinks as well as use of nicotine in quantities that may have reduced the effectiveness of neuroleptic drugs, in addition to use of alcohol and illicit drugs. Again this is consistent with the relatively low use of drugs in the Greek population [24].

However, these low levels of misconduct may be contrasted by the high level of stigma for patients with schizophrenia that have been reported in recent surveys of the Greek population. Three-quarters of respondents to a survey on attitudes to schizophrenia considered that such patients were a danger to the public because of violent behaviour. While it is possible that media presentation of isolated unprovoked violence perpetrated by the small minority of persistently offensive patients may have contributed to such attitudes, it is also possible that the close-knit family ties foster high tolerance of misconduct. This would suggest that our data may be biased by the unwillingness of family members to report episodes of misconduct towards them or other close associates.

Regardless of the absolute levels of misconduct and arguments about concealment and tolerance, the most striking finding we report is the substantial decrease in the levels of misconduct over the five years studied. At the last assessment only 3 (6%) patients showed persisting patterns of aggression, compared to 22% at the start of a project that ensured that optimal biomedical and psychosocial treatment was provided to the cohort. This treatment program was oriented to help patients and their caregivers improve the quality of their lives as well as attempting to eliminate all residual symptoms and problems, including substance abuse, lack of intimate relationships and aggressive behaviour. It seems reasonable to hypothesize that a comprehensive biopsychosocial treatment programme such as this may contribute to a lowered rate of misconduct. To date only the use of neuroleptic drugs in acute psychotic episodes has been associated with a reduction in aggressive behaviours [20,21]. The one-year follow-up study of Steadman and colleagues [15] showed that patients' levels of misconduct were elevated significantly more than their non-patient neighbours only in the first 10 weeks after discharge from hospital. Such a feature might suggest that community treatment was effective, but it may have also indicated that many patients were still highly symptomatic after relatively brief hospital treatment.

The three cases of persistent misconduct were all young single males living with their parents, who were highly stressed by their care. One merely shouted and threatened frequently without physical aggression. He had persistent delusions and negative symptoms as well as a paranoid personality disorder. The second developed schizophrenia aged 13, did not continue schooling and spent much of his adolescence in hospital. He had schizo-affective features of persistent psychotic symptoms with minimal negative symptoms, persistent generalized anxiety and frequent depressive episodes. He smoked cigarettes and drank alcohol regularly. The third had paranoid schizophrenia with persistent delusions and an antisocial personality disorder. His schizophrenic symptoms, social disability and cooperation with treatment improved over the three years and levels of unprovoked violence became much less frequent. These cases are consistent with epidemiological findings that associate antisocial behaviour patterns with persisting psychotic symptoms, personality abnormalities and substance use. It has been hypothesized that intensive cognitive behavioural treatments of the kinds used throughout this project applied at an early stage, perhaps before the onset of overt psychotic symptoms, may prevent criminal misbehaviour in this select group of difficult to manage cases [17]. The present study does not allow us to draw any constructive conclusions about the management of this specific subgroup, although all three showed some improvements in their antisocial behaviours with time.

The hypothesis that optimal treatment reduces levels of social misconduct including verbal, physical and sexual aggression is supported by our study. This finding is not new, but previous reports have focused largely on taking anti-psychotic medication on a regular basis. Optimal pharmacotherapy alone would fail to explain the continued improvement over 4 years of an intensive rehabilitation programme based on the implementation of evidence-based pharmacological and psychosocial treatment strategies in a well integrated goal-oriented system of continued care. Although cooperation with every aspect of the treatment programme was not always enthusiastic, there were no drop-outs over the four years of the project, even in cases where progress was minimal. In all cases the carers, usually family members, were included in the treatment team. Their personal concerns and goals were considered crucial, in particular when aggressive and antisocial behaviour by the patients affected their own well-being. Strategies for managing anger and frustration and the ability to communicate unpleasant feelings or desires in a non-threatening problem oriented way was at the basis of continued family interventions [25]. This as well as the introduction of innovative medications may have contributed to the decline in the severity and frequency of misconduct both at home and in the community.

Further research is needed so that community care for the seriously mentally disordered can be supported in a rational manner. In the absence of clearer predictors of antisocial acts, there is a strong tendency for mental health services to take a conservative view and to provide custodial care for the majority of cases in order to prevent the unacceptable acts of a small minority. As a recent reviewer of the links between mental illness and violence concluded "The challenge that lies ahead is one of further specifying the form of the relationship of mental illness and community violence and testing theories of how this relationship can differ across subgroups of mentally ill persons. Uncovering a broad relationship does not in itself promote sounder policy or more effective services. However, continued integration of research and service provision sensitive to what this relationship means in the lives of people with mental illness could move us toward this goal" [26].

## Conclusion

Aggressive and sexual misconduct is present in a minority of patients with schizophrenic disorders, and is commonly directed towards family members and friends. Comprehensive continued treatment with evidence-based biomedical and psychosocial treatment is associated with a reduction in this misconduct. However, a small group of persistent offenders characterised by continuous psychotic symptoms, personality disorders and substance use improve but may require more specialised or intensive interventions.

The small (but complete) inner city cohort that was studied prevented us from conducting correlational analyses of predictors of persistent misconduct. A larger cohort from the collaborative Optimal Treatment Project may provide predictive data in future reports.

## Competing Interests

The author(s) declare that they have no competing interests.

## References

[B1] Angermeyer MC Schizophrenia and violence Acta Psychiatr Scand 2000 Suppl 102 63 67 10.1034/j.1600-0447.2000.00012.x 11261644

[B2] Arseneault L Moffitt TE Caspi A Taylor PJ Silva PA Mental disorders and violence in a total birth cohort: results from the Dunedin Study Arch Gen Psychiatry 2000 57 979 86 10.1001/archpsyc.57.10.979 11015816

[B3] Hodgins S Epidemiological investigations of the associations between major mental disorders and crime: methodological limitations and validity of the conclusions Soc Psychiatry Psychiatr Epidemiol 1998 Suppl 33 29 37 10.1007/s001270050207 9857777

[B4] Modestin J Ammann R Mental disorders and criminal behaviour Br J Psychiatry 1995 166 667 675 7620755 10.1192/bjp.166.5.667

[B5] Swanson J Holzer C Ganju V Jono R Violence and psychiatric disorder in the community: evidence from the epidemiological catchment area surveys Hosp Community Psychiatry 1990 41 761 770 2142118 10.1176/ps.41.7.761

[B6] Volavka J Laska E Baker S Meisner M Czobor P Krivelevich I History of violent behaviour and schizophrenia in different cultures. Analyses based on the WHO study on Determinants of Outcome of Severe Mental Disorders Br J Psychiatry 1997 171 9 14 9328487 10.1192/bjp.171.1.9

[B7] Walsh E Leese M Taylor P Johnston I Burns T Creed F Higgit A Murray R Psychosis in high-security and general psychiatric services: report from the UK700 and special hospitals' treatment resistant schizophrenia groups Br J Psychiatry 2002 180 351 57 10.1192/bjp.180.4.351 11925359

[B8] Mullen PE A reassessment of the link between mental disorder and violent behaviour, and its implications for clinical practice Aust N Z J Psychiatry 1997 31 3 11 9088480 10.3109/00048679709073793

[B9] Rasmussen K Levander S Schizophrenia and violence Tidsskr Nor Laegeforen 2002 122 2303 5 12448274

[B10] Swartz MS Swanson JW Hiday VA Borum R Wagner HR Burns BJ Violence and severe mental illness: The effects of substance abuse and non-adherence to medication Am J Psychiatry 1998 155 226 31 9464202 10.1176/ajp.155.2.226

[B11] Eronen M Angermeyer MC Schulze B The psychiatric epidemiology of violent behaviour Soc Psychiatry Psychiatr Epidemiol 1998 33 13 23 10.1007/s001270050205 9857775

[B12] Walsh E Buchanan A Fahy T Violence and schizophrenia: examining the evidence Br J Psychiatry 2002 180 490 5 10.1192/bjp.180.6.490 12042226

[B13] Wessely SC Castle D Douglas AJ Taylor PJ The criminal careers of incident cases of schizophrenia Psychol Med 1994 2 483 502 10.1017/s0033291700027458 8084943

[B14] Taylor PJ When symptoms of psychosis drive serious violence Soc Psychiatry Psychiatr Epidemiol 1998 33 847 854 10.1007/s001270050209 9857779

[B15] Steadman HJ Mulvey EP Monahan J Violence by people discharged from acute psychiatric facilities and by others in the same neighborhoods Arch Gen Psychiatry 1998 55 393 401 10.1001/archpsyc.55.5.393 9596041

[B16] Hodgins S Hiskoke UL Freese R The antecedents of aggressive behavior among men with schizophrenias: a prospective investigation of patients in community treatment Behav Sci Law 2003 21 523 46 10.1002/bsl.540 12898506

[B17] Hodgins S Muller-Isberner R Preventing crime by people with schizophrenia: the role of psychiatric services Br J Psychiatry 2004 185 245 50 10.1192/bjp.185.3.245 15339830

[B18] Brekke JS Prindle C Bae SW Long JD Risks for individuals with schizophrenia who are living in the community Psychiatr Serv 2001 52 1281 10.1176/appi.ps.52.10.1358 11585953

[B19] Marley JA Buila S Crimes against people with mental illness: types, perpetrators, and influencing factors Social Work 2001 46 115 124 11329641 10.1093/sw/46.2.115

[B20] Chengappa KN Vasile J Levine J Ulrich R Baker R Gopalani A Schooler N Clozapine: its impact on aggressive behavior among patients in a state psychiatric hospital Schizophr Res 2002 53 1 6 10.1016/S0920-9964(00)00175-4 11728832

[B21] Steinert T Sippach T Gebhardt RP How common is violence in schizophrenia despite neuroleptic treatment? Pharmacopsychiatry 2000 33 98 102 10.1055/s-2000-342 10855460

[B22] Mullen P Violence and mental disorder Br J Hosp Med 1988 40 460 463 3067818

[B23] Falloon IRH Montero I Sungur M Mastroeni A Malm U Implementation of evidence-based treatment for schizophrenic disorders: two-year outcome of an international field trial of optimal treatment World Psychiatry 2004 3 104 9 PMC1414683 16633471

[B24] Kokkevi A Loukadakis M Plagianakou S Politikou K Stefanis C Sharp increase in illicit drug use in greece: trends from a general population survey on licit and illicit drug use Eur Addict Res 2000 6 42 9 10.1159/000019008 10729742

[B25] Falloon IRH OTP Collaborative Group Integrated mental health care: a guidebook for consumers and their carers Perugia: ARIETE 1997

[B26] Mulvey EP Assessing the evidence of a link between mental illness and violence Hosp Community Psychiatry 1994 45 663 8 7927290 10.1176/ps.45.7.663

